# Predictive Factors of Local Recurrence after Colorectal Cancer Liver Metastases Thermal Ablation

**DOI:** 10.3390/jimaging9030066

**Published:** 2023-03-10

**Authors:** Julien Odet, Julie Pellegrinelli, Olivier Varbedian, Caroline Truntzer, Marco Midulla, François Ghiringhelli, David Orry

**Affiliations:** 1 Radiology and Medical Imaging, Dijon Teaching Hospital, 21000 Dijon, France; 2 Radiology and Medical Imaging, Georges François Leclerc Cancer Center, 21000 Dijon, France; 3 Biostatistics and Bioinformatics, Georges François Leclerc Cancer Center, 21000 Dijon, France; 4 Pole of Medical Oncology, Georges François Leclerc Cancer Center, 21000 Dijon, France; 5 Pole of Surgical Oncology, Georges François Leclerc Cancer Center, 21000 Dijon, France

**Keywords:** thermoablation, microwave, radiofrequency, colorectal cancer, liver metastases, local recurrence

## Abstract

Background: Identify risk factors for local recurrence (LR) after radiofrequency (RFA) and microwave (MWA) thermoablations (TA) of colorectal cancer liver metastases (CCLM). Methods: Uni- (Pearson’s Chi^2^ test, Fisher’s exact test, Wilcoxon test) and multivariate analyses (LASSO logistic regressions) of every patient treated with MWA or RFA (percutaneously and surgically) from January 2015 to April 2021 in Centre Georges François Leclerc in Dijon, France. Results: Fifty-four patients were treated with TA for 177 CCLM (159 surgically, 18 percutaneously). LR rate was 17.5% of treated lesions. Univariate analyses by lesion showed factors associated with LR: sizes of the lesion (OR = 1.14), size of nearby vessel (OR = 1.27), treatment of a previous TA site LR (OR = 5.03), and non-ovoid TA site shape (OR = 4.25). Multivariate analyses showed that the size of the nearby vessel (OR = 1.17) and the lesion (OR = 1.09) remained significant risk factors of LR. Conclusions: The size of lesions to treat and vessel proximity are LR risk factors that need to be considered when making the decision of thermoablative treatments. TA of an LR on a previous TA site should be reserved to specific situations, as there is an important risk of another LR. An additional TA procedure can be discussed when TA site shape is non-ovoid on control imaging, given the risk of LR.

## 1. Introduction

Colorectal cancer is the second leading cause of cancer death worldwide. Approximately 50% of colorectal cancer patients will develop liver metastases. Surgery clearly improves prognosis, with 5-year overall survival rates (OS) of approximately 31–60% [1,2,3], but only 20% of patients are eligible. Thermoablation procedures increase the number of patients amenable to curative treatment. In early studies, local recurrence rates after thermal ablation were reported to be between 31 and 60% [4,5,6] and now range from 4 to 48% [7,8,9,10,11,12,13,14,15,16,17,18]. OS rates similar to those achieved with surgery are now observed with thermal ablation when treated lesions are <3 cm. Moreover, thermoablations yield a lower ablation volume of healthy hepatic parenchyma, with “parenchymal sparing”, lower complication rates, and a shorter procedural time and length of stay than with hepatic surgery [19,20,21,22]. The complementarity of thermoablation with surgical resection procedures is now well-established.

Some predictive factors of local recurrence have been identified, such as the size and localization of the lesion, or an ablation margin of less than 0.5 cm [23,24]. Proximity to large vessels is a risk factor for Radiofrequency Ablations (RFA) [25,26,27,28] but not for Microwave Ablations (MWA) [11,23,24]. Few studies have focused specifically on finding factors associated with local recurrence following thermoablation of colorectal cancer liver metastases.

Therefore, the aim of this study was to identify predictive factors of local recurrence following thermoablation of colorectal cancer liver metastases.

## 2. Materials and Methods

All patients who underwent thermoablation of colorectal cancer liver metastases during percutaneous or peri-operative procedures from 1 January 2015 to 1 April 2021 were retrospectively identified using the local computer informatics database in the Georges François Leclerc Cancer Center in Dijon, France. All patients had at least a 1 year follow-up after thermoablation and routine imaging follow-up at 3 months and 1 year. All treatment decisions were approved in multidisciplinary meetings.

### 2.1. Lesion Characteristics

Size, localisation (segment according to the Couinaud classification), distance to Glisson’s capsule, shortest distance to vessels whose diameter was greater than 3 mm, and the origin of the vessel in proximity (hepatic vessels, portal vessels, inferior vena cava…) were identified by pre-procedure cross-sectional imaging (pre and post-contrast CT-scan with late arterial and portal phases, pre and post-contrast MRI with dynamic injection up to 5 min and diffusion weighted imaging sequences).

### 2.2. Thermoablation Procedures

Percutaneous procedures were performed under general anaesthesia by 4 different radiologists with experience ranging from 8 to 22 years. The lesion(s) to be eradicated were found using a lesion-tracking CT-scan immediately prior to the procedure. In aseptic conditions, a needle topped with an electrode delivering the current was inserted into the lesion under CT controls for accurate placement. A post-procedure CT-scan was performed in each patient to ensure technical success was achieved with no residual tumour, covering the lesion to treat and a 1 cm margin manually measured in 3D side-by-side juxtaposition.

Surgical procedures were performed by a single surgeon with 15 years’ experience, during open or coelioscopic surgery. The choice between RFA and MWA was made considering the number of lesions to be treated and vessel proximity on pre-thermoablation imaging: MWA was chosen if there were more than 3 lesions to be treated or if the lesions were close to hepatic veins. The needle was inserted under ultrasound control. Ultrasound control was performed immediately after the procedure to make sure no residual tumour was left on site.

RFA was performed using a 20 or 30 mm Cool Tip^TM^ RF Ablation Single Electrode System E Series (MEDTRONIC, Minneapolis, MN, USA). RFA heat duration was 6′ with a 20 mm needle and 12′ with a 30 mm needle. Heat duration, electrode length and temperature at the end of procedure were recorded.

MWA was performed using an Emprint^TM^ Ablation System with 13-gauge antennas (MEDTRONIC, Minneapolis, MN, USA) at a frequency of 2.45 GHz. Power and heat duration of MWA were determined by a lesion size chart. Heat duration and power applied (Watts) were recorded.

Every thermoablation procedure was performed with a single needle. Use of Pringle manoeuvre and associated surgical resections were recorded for surgical approaches.

Follow-up liver MRI was performed using the same protocol, CT-scans explored thoracic, abdominal, and pelvic regions as part of the standard oncological follow-up, pre- and post-contrast with late arterial and venous portal phases on the abdomen. Thermoablation site aspect and shape were assessed at 1 month, 3 months, 6 months, 1 year, and every further 6 months. On follow-up imaging, the appearance of a nodule contiguous to the edges of the thermoablation site was considered as a local recurrence.

We calculated the difference between the sizes (length and width) of the thermoablation area and the lesion treated at 3-months and 1-year imaging. Measures were carefully drawn by a radiologist with 4 years’ experience. Two radiologists (one with 4 years and another with 8 years’ experience) recorded local recurrence.

### 2.3. Statistical Analyses

Statistical analyses were performed using “R” software for statistical computing.

Parameters were compared by recurrence status by univariate analyses using the Chi square or Fisher’s exact test for qualitative variables, and the Wilcoxon test for continuous variables, as appropriate. Multivariate analyses were performed including variables associated with recurrent lesions chosen by LASSO logistic regression [29]. Statistical tests were two-sided, and a *p*-value of <0.05 was considered statistically significant.

Analyses were performed using R statistical software, R-4.2.2, (R Core Team 2017).

Primary outcome was thermoablation site local recurrence during follow-up.

## 3. Results

### Patient Characteristics

Fifty-four patients met the inclusion criteria in 69 ablation sessions: 42 patients had only one thermoablation procedure, 10 had two procedures, one had three, and one had four. The patient and disease characteristics are detailed in Table 1 and Table 2.

A total of 177 lesions were treated: 118 lesions was treated by RFA, 59 by MWA. One hundred and fifty-nine lesions were treated by the surgical approach (158 by open surgery, 1 by coelioscopic surgery) and 18 by the percutaneous approach. The mean number of lesions treated per patient was 3.3. Among the lesions, nine lesions were retreated lesions.

No patient had grade IV–V complications, 2% of patients had grade III complications. No patient had biliary complications such as biliary stenosis or fistula. No visual residual tumour was found during post-procedure imaging.

No patient had local recurrence at 1-month control.

Local recurrence occurred in 31 of 177 lesions (17.5%). The median time to local recurrence was 377 days [158;702]. The primary efficacy rate was 84.5%, as local recurrence occurred in 26 out of the 168 lesions initially treated. The secondary efficacy rate was 82.5%, as local recurrence occurred in five out of the nine retreated lesions.

**Figure 1:**
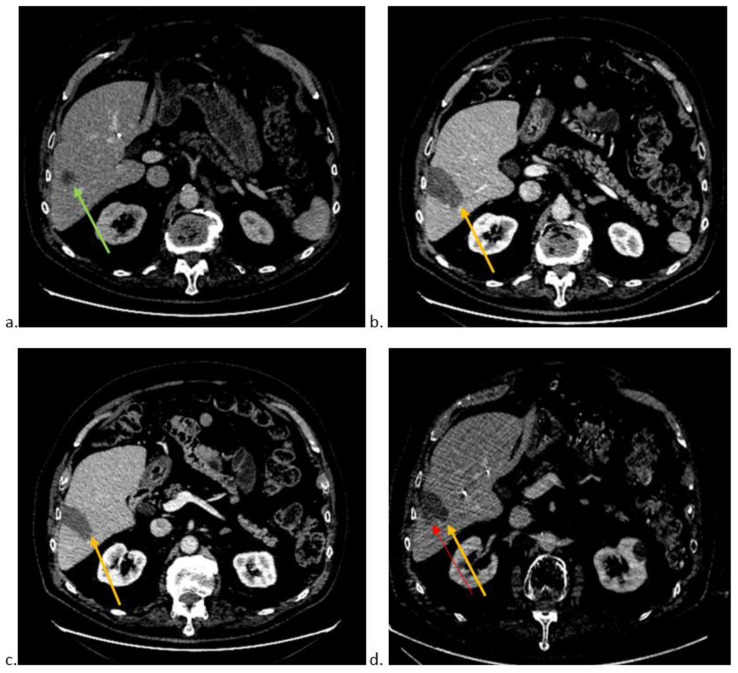
(**a**). Axial CT-scan image at portal phase showing colorectal liver metastasis (green arrow) in segment VI. (**b**). Axial CT-scan image at portal phase showing technical success of thermoablation (orange arrow) with no residual tumour. (**c**). Axial CT-scan image at portal phase of the lowest part of thermoablation area (orange arrow). (**d**). Axial CT-scan image at portal phase showing local recurrence (red arrow) at the edge of the thermoablation site (orange arrow).

Figure 1 shows an example of local recurrence.

There was no significant difference in the rate of local recurrence between liver segments (*p* = 0.35). However, the local recurrence rate was 50% in segment I.

The comparison of lesion characteristics by recurrence status is detailed in Table 3.

There was no significant difference between thermoablation site sizes and expected sizes according to the charts.

By univariate analysis (Table 4 and Table 5), patient, lesion, and procedure related factors favouring local recurrence incurred:

- mild risk: total number of chemotherapy lines (OR = 1.69), size of nearby vessel (OR = 1.27), sizes of the metastasis (OR = 1.11 for length and 1.14 for width);
- moderate risk: extrahepatic lesions at the time of thermoablation (OR = 2.48), number of chemotherapy lines before thermoablation (OR = 2.17), percutaneous approach (OR = 3.97);
- high risk: treatment of a local recurrence on previous thermoablation site (OR = 5.03), treatment of a liver subject to metastatic recurrence (OR = 5.16), non-ovoid thermoablation site shape (OR = 4.25).

The thermoablation margin’s width and the distance to a large vessel (size > 3 mm) were associated with a lower recurrence risk.

Multivariate analysis was performed on variables chosen by LASSO logistic regression statistical computing (Table 6).

The size of the lesion and the size of the nearby vessel remained significant (OR = 1.09 and 1.17, respectively).

When focusing on vessels, it was found that thermoablation next to hepatic veins was statistically associated with local recurrence compared to other vessels (*p* = 0.001), as 12 lesions out of 30 had local recurrence next to hepatic veins versus 19 out of 128 for other vessels.

The shape of the thermoablation site was classified into ovoid/discoid and others (pyriform, bilobed…). No statistical difference in terms of shape was found between RFA and MWA, but there was a higher rate (*p* = 0.03) of non-ovoid shapes in the percutaneous approach (8/17) than in the surgical approach (33/158).

## 4. Discussion

Imaging modalities such as CT and MRI play a central role in the management of colorectal liver metastases, from diagnosis to treatment, and even afterwards, to track local recurrence.

This study suggests that many factors can affect local recurrence.

Patient characteristics showed that a broad range of lesions underwent thermoablation, with a mean number of 3.3 lesions treated per patient and some patients undergoing up to 9 thermoablations in the same procedure. Recurrence rates found in our study are similar to results found in the literature [7,8,9,10,11,12,13,14,15,16,17,18].

Patient-related characteristics were not significantly associated with local recurrence in our study. Therefore, the results were not affected by patient bias and refer to lesions only. Our study complied with the recommendations on standard criteria such as lesion size < 3 cm. The comparison between the size of the thermoablation site and the expected size on the charts showed no significant difference. Nevertheless, lesion sizes, distance to, and size of nearby vessels > 3 mm, even more so if they were hepatic veins, and non-ovoid thermoablation site shape were all associated with local recurrence by univariate analysis and may account for some untreated areas of the metastasis. The size of the lesion and the proximity of vessels are well-known risk factors for local recurrence [23,25,26,27,28,30]. They also appeared to be associated with local recurrence in multivariate analyses. A short distance to nearby vessels was found to be a risk factor even for MWA.

The novel information provided by this study is that non-ovoid thermoablation site shape is related to local recurrence by univariate analysis and was borderline significant in multivariate analysis. “Heat Sink Effect” may be responsible for this shape and can explain why it is related to local recurrence, with suboptimal and unequal delivery of the heat. This hypothesis is consistent with the results of a study conducted within in vivo porcine models [31], showing that the shape of the thermoablation site is influenced by intrahepatic vessels. It is known that the proximity of blood vessels greater than 3 mm leads to the “Heat Sink Effect”, essentially affecting RFA because of the conduction-based heating spreading in this technique. It is caused by blood irrigation cooling the tissue in the local area surrounding the vessel, therefore, preventing completion of the total theoretical thermoablation area. It can be removed in surgical procedures by clamping the portal pedicle (Pringle Manoeuvre) when the lesion is next to a portal branch or by parenchymal compressing for lesions next to hepatic veins [32]. MWA, lying on dielectric permittivity instead of conduction, is less affected by the “Heat Sink Effect” [32,33,34]. The “Heat Sink Effect” could explain the high local recurrence rate found in segment I (50%) because of the proximity of the inferior vena cava and the portal pedicle. The absence of statistical significance may be due to a lack of power, as there were only four lesions treated in this segment.

Therefore, this study suggests that the shape of the ablation area after the procedure should be examined, as there is a higher risk of local recurrence when non-ovoid. It raises the discussion of additional ablation, which could be provided by replacing the needle or by inserting a second or even a third needle to better control the thermoablation area. Irreversible Electroporation [35,36,37,38] and High-Intensity Focused Ultrasounds [39,40] are alternative ablation techniques for such lesions, their efficacy and safety have been demonstrated in many studies especially for colorectal liver metastases treatment.

Our secondary efficacy rate results showed that thermoablation of a local recurrence on a previous thermoablation site is a risk factor for a new local recurrence. The risk factors due to the localisation of the initial metastasis (vessel proximity…) may have caused the first local recurrence. As they remain the same from one thermoablation to another at the same site, a higher risk of local recurrence was expected and observed.

The size difference between the thermoablation site and the lesion’s edges was calculated to represent the security margin. Greater security margins were associated with a lower recurrence rate in our study, significantly for the width, and almost significant for the length. Shady et al. [7] demonstrated that a 1 cm security margin was associated with almost no local recurrence. Currently, software with image matching and margin estimations is being developed and examined as a future prospect to help deal with the security margins in a simpler way. Manual detection and segmentation of colorectal liver metastases by radiologists can be assisted by deep learning automated detection and segmentation of liver lesions as it has proven its efficiency [41]. Radiomic approaches have been shown to be very helpful for liver lesion segmentation, given lesion heterogeneity, and should be integrated as future clinical prospects in this field of oncoradiology [42]. Texture-based features could be part of procedure planning, but may also be of interest for detecting local recurrence as biomarkers [43,44].

Some limitations of our study are inherent to its design: single-centred with a relatively low number of patients and heterogeneity in treatments’ details (surgical versus percutaneous, MWA versus RFA).

Our results comparing MWA and RFA, but also surgical versus percutaneous approaches, likely suffered from selection bias. Indeed, the type of thermoablation to be used was chosen before the procedure, knowing that a high number of lesions to be treated or lesions next to vessels prompted clinicians to use MWA more than RFA. In short, the patients chosen to be treated with MWA had more hepatic lesions to treat than those treated with RFA. Furthermore, MWA was preferred for hepatic lesions next to vessels, and were therefore more at risk of local recurrence. Similar to our study, other studies in the literature have suggested that the percutaneous approach is associated with a higher local recurrence rate than the surgical approach [6,30,45,46,47,48]. However, the analysis comparing the two approaches could be biased.

It is widely acknowledged that patients undergoing other surgical resections during the same procedure as thermoablation are subject to a higher level of inflammatory factors, which could lead to a burst of potential local tumour residue. This was not the case in our study.

Thermoablation procedures appeared to be safe with a low complication rate: only 2% of patients had grade III complications, no patient had grade IV complications. No patient had biliary complications such as biliary stenosis or fistula.

Genetic mutations were not statistically involved in local recurrence in our study, but some other studies have found a link between these genetic mutations with an adverse impact on oncological outcomes [49,50].

## 5. Conclusions

Thermoablation is a safe procedure and is efficient in terms of parenchymal sparing, with a low complication rate. Precise mapping of the lesion and its local environment can help identify factors that may be responsible for treatment inefficiency. Larger size of the lesion to treat and proximity to blood vessels (especially hepatic veins) are risk factors for local recurrence that need to be considered when deciding on the ablation strategy, by adjusting the number of needles and the heating parameters to the lesion to treat or by taking a different approach with non-thermal ablations. Thermoablation of local recurrence occurring at a previous thermoablation site should be reserved for specific situations, since there is a higher risk of further local recurrence, given the almost identical primary and secondary efficacy rates, and this should also be balanced with alternative ablation techniques (IRE, HIFU) [35,36,37,39]. A non-ovoid thermoablation site shape on routine control imaging should prompt consideration of an early additional thermoablation procedure given the likelihood of a suboptimal distribution of the heat and the risk of a subsequent local recurrence.

As surgical procedures use US guidance, and cross-sectional images are not immediately available before the end of the procedure to confirm technical success. Contrast ultrasonography could be encouraged during surgery to offer arguments for whole lesion destruction, and, if destruction is not complete, to provide immediate additional treatment.

As future prospects, developments in the fields of imaging and technology will enable treatment plans tailored to each individual and lesion, with their own local recurrence risk factors [51]. Thermoablation charts could also take local recurrence risk factors into account, identified upstream on pre-procedure imaging, to adapt the thermoablation parameters and make them appropriate to the lesion to be treated. Modelling software could give us an overview of what the thermoablation area will look like, depending on the needle’s position, given the local risk factors and, especially, vessel proximity. Therefore, it could enable us to select the best path to treat the lesion [52,53].

## Figures and Tables

**Table 1:** Patient characteristics.

Characteristics	N = 69 ^1^
Gender Male Female	49 (71%) 20 (29%)
BMI (kg·m^−2^)	26.0 (23.4–28.3)
WHO performance status 0 1 2	53 (76.8%) 15 (21.7%) 1 (1.5%)
Age at primary cancer diagnosis (years)	65 (57–70)
Primary cancer site Left colon Right colon Rectum	26 (37.7%) 17 (24.6%) 26 (37.7%)
Chemotherapy prior to primary cancer surgery Yes No	36 (52%) 33 (48%)
CEA (µg/L) Unknown	3 (2–13) 4
CA19-9 (U/mL) Unknown	12 (8–30) 4
Extrahepatic secondary lesions at the time of thermoablation No Yes	40 (58%) 29 (42%)
Colorectal TNM:T 1 2 3 4	0 (0%) 4 (6%) 46 (67%) 19 (27%)
Colorectal TNM:N 0 1 2	11 (16%) 45 (66%) 10 (18%)
Genetics RAS mutation RAS Wild type BRAF mutation BRAF Wild type MSI phenotype MSS phenotype	26 (38%) 43 (62%) 6 (9%) 63 (91%) 2 (2.9%) 67 (97%)

^1^ Number of sessions (%); Median (IQR).

**Table 2:** Liver disease characteristics.

Characteristics	N = 69 ^1^
Hepatic metastases chronological occurrence Synchronous Metachronous	41 (59%) 28 (41%)
Interval between primary cancer diagnosis and metachronous metastases occurrence (days)	694 (454–1058)
Hepatic metastatic recurrence Yes No	21 (30%) 48 (70%)
Metastases preoperative chemotherapy Yes No	66 (96%) 3 (4%)
Number of chemotherapy lines prior to thermoablation	1 (1–2)
Total number of chemotherapy lines	3 (2–4)
Underlying liver condition None Steatosis Child A6 cirrhosis	30 (43.5%) 38 (55%) 1 (1.5%)
Number of liver metastases to be treated by episode (thermoablation only)	2 (1–3)
Number of liver metastases to be treated by episode (thermoablation + surgery)	3 (2–6)
Treatment of a previous thermoablation site recurrence Yes No	9 (13%) 60 (87%)

^1^ Number of sessions (%); Median (IQR).

**Table 3:** Comparison of lesion characteristics by recurrence status.

Characteristics	No Recurrence (N = 146 ^1^)	Recurrence (N = 31 ^1^)	*p*-Value ^2^
Metastases’ chronological occurrence Synchronous Metachronous	100 (68%) 46 (32%)	17 (55%) 14 (45%)	0.14
Hepatic metastatic recurrence	25 (17%)	16 (52%)	<0.001
Metastases preoperative chemotherapy	142 (97%)	31 (100%)	>0.9
Number of chemotherapy lines prior to thermoablation	1 (1–2)	2 (1–3)	0.001
Total number of chemotherapy lines	3 (2–4)	4 (3–4)	0.002
Underlying liver condition None Others (steatosis, cirrhosis)	55 (38%) 91 (62%)	15 (48%) 16 (52%)	0.3
Number of liver metastases to be treated with thermoablation by episode	3 (2–6)	2 (2–4)	0.009
Treatment of a previous thermoblation site reccurence Yes No	8 (5.5%) 138 (94.5%)	7 (23%) 24 (77%)	0.006
Thermoablation type Radiofrequency ablation Microwave ablation	102 (70%) 44 (30%)	16 (52%) 15 (48%)	0.050
Thermoablation approach Surgical Percutaneous	135 (92.5%) 11 (7.5%)	24 (77%) 7 (23%)	0.020
Guidance during procedure Ultrasonography CT scan	136 (93.2%) 10 (6.8%)	25 (81%) 6 (19%)	0.039
Pringle Manoeuvre	13 (8.9%)	1 (3.2%)	0.5
Procedure complication grade (Clavien–Dindo classification) 0 1 2 3 Unknown	78 (58%) 40 (29%) 15 (11%) 3 (2%) 10	13 (52%) 11 (44%) 1 (4%) 0 (0%) 6	0.5
Hepatic segment I II III IV V VI VII VIII	2 (50%) 12 (86%) 9 (100%) 16 (89%) 20 (77%) 18 (78%) 28 (90%) 41 (78%)	2 (50%) 2 (14%) 0 (0%) 2 (11%) 6 (23%) 5 (22%) 3 (10%) 11 (22%)	0.14 1 0.36 0.74 0.41 0.56 0.3 0.51

^1^ n (%); Median (IQR); ^2^ Fisher’s exact test, Wilcoxon rank sum test, Pearson’s Chi-squared test.

**Table 4:** Relation between patient characteristics and local recurrence.

Characteristics	N	OR ^1^	95% CI ^1^	*p*-Value
Gender (male vs. female)	177	2.73	[0.99–9.64]	0.076
WHO performance status	177	0.73	[0.26–1.73]	0.5
BMI	177	0.98	[0.88–1.08]	0.6
CEA	168	1.00	[1.00–1.00]	0.7
CA19-9	168	0.99	[0.98–1.00]	0.3
Age at primary cancer diagnosis	177	0.99	[0.95–1.02]	0.5
Extrahepatic secondary lesions at the time of thermoablation	177	2.48	[1.12–5.52]	0.024
Genetics RAS wild type BRAF wild type MSS phenotype	177 177 177	1.14 0.69 1.06	[0.52–2.57] [0.20–3.20] [0.16–20.8]	0.8 0.6 >0.9
Metastases chronological occurrence	177	1.79	[0.80–3.94]	0.15
Hepatic metastatic recurrence	177	5.16	[2.27–11.9]	<0.001
Number of chemotherapy lines prior to thermoablation	177	2.17	[1.39–3.50]	<0.001
Total number of chemotherapy lines	177	1.69	[1.23–2.41]	0.002
Underlying liver condition	177	0.64	[0.29–1.42]	0.3
Number of metastases to be treated with thermoablation	177	0.79	[0.64–0.94]	0.016
Treatment of a previous thermoablation site	177	5.03	[1.63–15.3]	0.004

^1^ OR = Odds Ratio, CI = Confidence Interval.

**Table 5:** Relation between metastasis characteristics and local recurrence.

Characteristics	N	OR ^1^	95% CI ^1^	*p*-Value
Thermoablation type (MWA versus RFA)	177	2.17	[0.98–4.80]	0.054
Thermoablation approach (percutaneous versus surgical)	177	3.97	[1.33–11.4]	0.011
Heating Time (sec)	177	1.05	[0.94–1.19]	0.4
Power (MWA) (Watts)	59	1.02	[0.99–1.07]	0.2
Electrode size (RFA) (mm)	118	1.10	[0.99–1.23]	0.087
End of procedure temperature	118	1.09	[0.96–1.26]	0.2
Size of nearby vessel (mm)	177	1.27	[1.13–1.45]	<0.001
Distance to vessel with diameter > 3 mm	177	0.59	[0.44–0.78]	<0.001
Thermoablation site shape (non-ovoid versus ovoid)	177	4.25	[1.87–9.76]	<0.001
Metastasis length (mm)	177	1.11	[1.05–1.18]	<0.001
Metastasis width (mm)	177	1.14	[1.07–1.23]	<0.001
Thermoablation site length (mm)	177	1.02	[0.98–1.07]	0.3
Thermoablation site width (mm)	177	1.00	[0.95–1.06]	0.9
Thermoablation length margin (mm)	177	0.95	[0.89–1.00]	0.080
Thermoablation width margin (mm)	177	0.90	[0.83–0.96]	0.002

^1^ OR = Odds Ratio, CI = Confidence Interval.

**Table 6:** Relation between characteristics chosen by LASSO logistic regression and local recurrence.

Characteristics	N	OR ^1^	95% CI ^1^	*p*-Value
Treatment of a previous thermoablation site	177	2.47	[0.64–9.26]	0.2
Thermoablation approach (surgical versus percutaneous)	177	0.35	[0.10–1.30]	0.10
Metastasis width (mm)	177	1.09	[1.01–1.19]	0.029
Size of nearby vessel (mm)	177	1.17	[1.03–1.35]	0.025
Distance to vessel > 3 mm	177	0.45	[0.11–1.48]	0.2
Shape of thermoablation site (non-ovoid versus ovoid)	177	2.60	[0.97–6.80]	0.052

^1^ OR = Odds Ratio, CI = Confidence Interval.

## Data Availability

No new data were created or analyzed in this study.

## References

[1] Viganò L., Capussotti L., Majno P., Toso C., Ferrero A., De Rosa G., Rubbia-Brandt L., Mentha G. (2015). Liver Resection in Patients with Eight or More Colorectal Liver Metastases. Br. J. Surg..

[2] Imai K., Benitez C.C., Allard M.-A., Vibert E., Cunha A.S., Cherqui D., Castaing D., Bismuth H., Baba H., Adam R. (2015). Failure to Achieve a 2-Stage Hepatectomy for Colorectal Liver Metastases: How to Prevent It?. Ann. Surg..

[3] Ruers T., Van Coevorden F., Punt C.J.A., Pierie J.-P.E.N., Borel-Rinkes I., Ledermann J.A., Poston G., Bechstein W., Lentz M.-A., Mauer M. (2017). Local Treatment of Unresectable Colorectal Liver Metastases: Results of a Randomized Phase II Trial. J. Natl. Cancer Inst..

[4] de Baere T., Elias D., Dromain C., Din M.G., Kuoch V., Ducreux M., Boige V., Lassau N., Marteau V., Lasser P. (2000). Radiofrequency Ablation of 100 Hepatic Metastases with a Mean Follow-up of More than 1 Year. AJR Am. J. Roentgenol..

[5] Solbiati L., Livraghi T., Goldberg S.N., Ierace T., Meloni F., Dellanoce M., Cova L., Halpern E.F., Gazelle G.S. (2001). Percutaneous Radio-Frequency Ablation of Hepatic Metastases from Colorectal Cancer: Long-Term Results in 117 Patients. Radiology.

[6] Kuvshinoff B.W., Ota D.M. (2002). Radiofrequency Ablation of Liver Tumors: Influence of Technique and Tumor Size. Surgery.

[7] Shady W., Petre E.N., Gonen M., Erinjeri J.P., Brown K.T., Covey A.M., Alago W., Durack J.C., Maybody M., Brody L.A. (2016). Percutaneous Radiofrequency Ablation of Colorectal Cancer Liver Metastases: Factors Affecting Outcomes—A 10-Year Experience at a Single Center. Radiology.

[8] Leung U., Kuk D., D’Angelica M.I., Kingham T.P., Allen P.J., DeMatteo R.P., Jarnagin W.R., Fong Y. (2014). Long-Term Outcomes Following Microwave Ablation for Liver Malignancies. Br. J. Surg..

[9] Kose E., Kahramangil B., Aydin H., Donmez M., Takahashi H., Aucejo F., Siperstein A., Berber E. (2020). Outcomes of Laparoscopic Tumor Ablation for Neuroendocrine Liver Metastases: A 20-Year Experience. Surg. Endosc..

[10] North D.A., Groeschl R.T., Sindram D., Martinie J.B., Iannitti D.A., Bloomston M., Schmidt C., Rilling W.S., Gamblin T.C., Martin R.C.G. (2014). Microwave Ablation for Hepatic Malignancies: A Call for Standard Reporting and Outcomes. Am. J. Surg..

[11] Correa-Gallego C., Fong Y., Gonen M., D’Angelica M.I., Allen P.J., DeMatteo R.P., Jarnagin W.R., Kingham T.P. (2014). A Retrospective Comparison of Microwave Ablation vs. Radiofrequency Ablation for Colorectal Cancer Hepatic Metastases. Ann. Surg. Oncol..

[12] Swan R.Z., Sindram D., Martinie J.B., Iannitti D.A. (2013). Operative Microwave Ablation for Hepatocellular Carcinoma: Complications, Recurrence, and Long-Term Outcomes. J. Gastrointest. Surg. Off. J. Soc. Surg. Aliment. Tract.

[13] Martin R.C.G., Scoggins C.R., McMasters K.M. (2010). Safety and Efficacy of Microwave Ablation of Hepatic Tumors: A Prospective Review of a 5-Year Experience. Ann. Surg. Oncol..

[14] Kennedy T.J., Cassera M.A., Khajanchee Y.S., Diwan T.S., Hammill C.W., Hansen P.D. (2013). Laparoscopic Radiofrequency Ablation for the Management of Colorectal Liver Metastases: 10-Year Experience. J. Surg. Oncol..

[15] Machi J., Oishi A.J., Sumida K., Sakamoto K., Furumoto N.L., Oishi R.H., Kylstra J.W. (2006). Long-Term Outcome of Radiofrequency Ablation for Unresectable Liver Metastases from Colorectal Cancer: Evaluation of Prognostic Factors and Effectiveness in First- and Second-Line Management. Cancer J. Sudbury Mass.

[16] Hildebrand P., Kleemann M., Roblick U.J., Mirow L., Birth M., Leibecke T., Bruch H.-P. (2006). Radiofrequency-Ablation of Unresectable Primary and Secondary Liver Tumors: Results in 88 Patients. Langenbecks Arch. Surg..

[17] Berber E., Tsinberg M., Tellioglu G., Simpfendorfer C.H., Siperstein A.E. (2008). Resection versus Laparoscopic Radiofrequency Thermal Ablation of Solitary Colorectal Liver Metastasis. J. Gastrointest. Surg. Off. J. Soc. Surg. Aliment. Tract.

[18] Takahashi H., Berber E. (2020). Role of Thermal Ablation in the Management of Colorectal Liver Metastasis. Hepatobiliary Surg. Nutr..

[19] Berber E., Siperstein A.E. (2007). Perioperative Outcome after Laparoscopic Radiofrequency Ablation of Liver Tumors: An Analysis of 521 Cases. Surg. Endosc..

[20] Solbiati L., Ierace T., Goldberg S.N., Sironi S., Livraghi T., Fiocca R., Servadio G., Rizzatto G., Mueller P.R., Del Maschio A. (1997). Percutaneous US-Guided Radio-Frequency Tissue Ablation of Liver Metastases: Treatment and Follow-up in 16 Patients. Radiology.

[21] Puijk R.S., Ruarus A.H., Vroomen L.G.P.H., van Tilborg A.A.J.M., Scheffer H.J., Nielsen K., de Jong M.C., de Vries J.J.J., Zonderhuis B.M., COLLISION Trial Group (2018). Colorectal Liver Metastases: Surgery versus Thermal Ablation (COLLISION)—A Phase III Single-Blind Prospective Randomized Controlled Trial. BMC Cancer.

[22] Meijerink M.R., Puijk R.S., van Tilborg A.A.J.M., Henningsen K.H., Fernandez L.G., Neyt M., Heymans J., Frankema J.S., de Jong K.P., Richel D.J. (2018). Radiofrequency and Microwave Ablation Compared to Systemic Chemotherapy and to Partial Hepatectomy in the Treatment of Colorectal Liver Metastases: A Systematic Review and Meta-Analysis. Cardiovasc. Intervent. Radiol..

[23] Takahashi H., Kahramangil B., Berber E. (2018). Local Recurrence after Microwave Thermosphere Ablation of Malignant Liver Tumors: Results of a Surgical Series. Surgery.

[24] Takahashi H., Akyuz M., Aksoy E., Karabulut K., Berber E. (2017). Local Recurrence after Laparoscopic Radiofrequency Ablation of Malignant Liver Tumors: Results of a Contemporary Series. J. Surg. Oncol..

[25] Groeschl R.T., Pilgrim C.H.C., Hanna E.M., Simo K.A., Swan R.Z., Sindram D., Martinie J.B., Iannitti D.A., Bloomston M., Schmidt C. (2014). Microwave Ablation for Hepatic Malignancies: A Multiinstitutional Analysis. Ann. Surg..

[26] Petre E.N., Sofocleous C. (2017). Thermal Ablation in the Management of Colorectal Cancer Patients with Oligometastatic Liver Disease. Visc. Med..

[27] Berber E., Siperstein A. (2008). Local Recurrence After Laparoscopic Radiofrequency Ablation of Liver Tumors: An Analysis of 1032 Tumors. Ann. Surg. Oncol..

[28] Wong S.L., Mangu P.B., Choti M.A., Crocenzi T.S., Dodd G.D., Dorfman G.S., Eng C., Fong Y., Giusti A.F., Lu D. (2010). American Society of Clinical Oncology 2009 Clinical Evidence Review on Radiofrequency Ablation of Hepatic Metastases From Colorectal Cancer. J. Clin. Oncol..

[29] Tibshirani R. (1996). Regression Shrinkage and Selection via the LASSO. J. R. Stat. Soc. B.

[30] Mulier S., Ni Y., Jamart J., Ruers T., Marchal G., Michel L. (2005). Local Recurrence After Hepatic Radiofrequency Coagulation: Multivariate Meta-Analysis and Review of Contributing Factors. Ann. Surg..

[31] Frericks B.B., Ritz J.P., Albrecht T., Valdeig S., Schenk A., Wolf K.-J., Lehmann K. (2008). Influence of Intrahepatic Vessels on Volume and Shape of Percutaneous Thermal Ablation Zones: In Vivo Evaluation in a Porcine Model. Investig. Radiol..

[32] Rhaiem R., Kianmanesh R., Minon M., Tashkandi A., Aghaei A., Ledoux G., Hoeffel C., Bouche O., Sommacale D., Piardi T. (2020). Microwave Thermoablation of Colorectal Liver Metastases Close to Large Hepatic Vessels Under Pringle Maneuver Minimizes the “Heat Sink Effect”. World J. Surg..

[33] Bhardwaj N., Dormer J., Ahmad F., Strickland A.D., Gravante G., West K., Dennison A.R., Lloyd D.M. (2011). Microwave Ablation of the Liver: A Description of Lesion Evolution over Time and an Investigation of the Heat Sink Effect. Pathology.

[34] Vogl T.J., Nour-Eldin N.-E.A., Hammerstingl R.M., Panahi B., Naguib N.N.N. (2017). Microwave Ablation (MWA): Basics, Technique and Results in Primary and Metastatic Liver Neoplasms—Review Article. RoFo-Fortschr. Geb. Rontgenstr. Nuklearmed..

[35] Belfiore M.P., De Chiara M., Reginelli A., Clemente A., Urraro F., Grassi R., Belfiore G., Cappabianca S. (2022). An Overview of the Irreversible Electroporation for the Treatment of Liver Metastases: When to Use It. Front. Oncol..

[36] Koethe Y., Wilson N., Narayanan G. (2022). Irreversible Electroporation for Colorectal Cancer Liver Metastasis: A Review. Int. J. Hyperth..

[37] Tameez Ud Din A., Tameez-Ud-Din A., Chaudhary F.M.D., Chaudhary N.A., Siddiqui K.H. (2019). Irreversible Electroporation For Liver Tumors: A Review Of Literature. Cureus.

[38] Scheffer H.J., Melenhorst M.C.A.M., Echenique A.M., Nielsen K., van Tilborg A.A.J.M., van den Bos W., Vroomen L.G.P.H., van den Tol P.M.P., Meijerink M.R. (2015). Irreversible Electroporation for Colorectal Liver Metastases. Tech. Vasc. Interv. Radiol..

[39] Yang T., Chen Q., Kuang L., Fu Z., Wang Y., Chen Y., Yang L., Xu Y. (2022). Effectiveness and Safety of Ultrasound-Guided High-Intensity Focused Ultrasound Ablation for the Treatment of Colorectal Cancer Liver Metastases. Int. J. Hyperth..

[40] Militello C., Vitabile S., Rundo L., Russo G., Midiri M., Gilardi M.C. (2015). A Fully Automatic 2D Segmentation Method for Uterine Fibroid in MRgFUS Treatment Evaluation. Comput. Biol. Med..

[41] Vorontsov E., Cerny M., Régnier P., Di Jorio L., Pal C.J., Lapointe R., Vandenbroucke-Menu F., Turcotte S., Kadoury S., Tang A. (2019). Deep Learning for Automated Segmentation of Liver Lesions at CT in Patients with Colorectal Cancer Liver Metastases. Radiol. Artif. Intell..

[42] Rundo L., Beer L., Ursprung S., Martin-Gonzalez P., Markowetz F., Brenton J.D., Crispin-Ortuzar M., Sala E., Woitek R. (2020). Tissue-Specific and Interpretable Sub-Segmentation of Whole Tumour Burden on CT Images by Unsupervised Fuzzy Clustering. Comput. Biol. Med..

[43] Escudero Sanchez L., Rundo L., Gill A.B., Hoare M., Mendes Serrao E., Sala E. (2021). Robustness of Radiomic Features in CT Images with Different Slice Thickness, Comparing Liver Tumour and Muscle. Sci. Rep..

[44] Wang Y., Ma L.-Y., Yin X.-P., Gao B.-L. (2022). Radiomics and Radiogenomics in Evaluation of Colorectal Cancer Liver Metastasis. Front. Oncol..

[45] Choy P.Y.G., Koea J., McCall J., Holden A., Osbourne M. (2002). The Role of Radiofrequency Ablation in the Treatment of Primary and Metastatic Tumours of the Liver: Initial Lessons Learned. N. Z. Med. J..

[46] Jiao L.R., Hansen P.D., Havlik R., Mitry R.R., Pignatelli M., Habib N. (1999). Clinical Short-Term Results of Radiofrequency Ablation in Primary and Secondary Liver Tumors. Am. J. Surg..

[47] Poon R.T., Ng K.K., Lam C.M., Ai V., Yuen J., Fan S.T., Wong J. (2004). Learning Curve for Radiofrequency Ablation of Liver Tumors: Prospective Analysis of Initial 100 Patients in a Tertiary Institution. Ann. Surg..

[48] Lu D.S.K., Raman S.S., Limanond P., Aziz D., Economou J., Busuttil R., Sayre J. (2003). Influence of Large Peritumoral Vessels on Outcome of Radiofrequency Ablation of Liver Tumors. J. Vasc. Interv. Radiol. JVIR.

[49] Shady W., Petre E.N., Vakiani E., Ziv E., Gonen M., Brown K.T., Kemeny N.E., Solomon S.B., Solit D.B., Sofocleous C.T. (2017). Kras Mutation Is a Marker of Worse Oncologic Outcomes after Percutaneous Radiofrequency Ablation of Colorectal Liver Metastases. Oncotarget.

[50] Dijkstra M., Nieuwenhuizen S., Puijk R.S., Timmer F.E.F., Geboers B., Schouten E.A.C., Opperman J., Scheffer H.J., de Vries J.J.J., Versteeg K.S. (2021). Primary Tumor Sidedness, RAS and BRAF Mutations and MSI Status as Prognostic Factors in Patients with Colorectal Liver Metastases Treated with Surgery and Thermal Ablation: Results from the Amsterdam Colorectal Liver Met Registry (AmCORE). Biomedicines.

[51] Hoffer E.K., Borsic A., Patel S.D. (2022). Validation of Software for Patient-Specific Real-Time Simulation of Hepatic Radiofrequency Ablation. Acad. Radiol..

[52] Luo M., Jiang H., Shi T. (2022). Multi-Stage Puncture Path Planning Algorithm of Ablation Needles for Percutaneous Radiofrequency Ablation of Liver Tumors. Comput. Biol. Med..

[53] Reinhardt M., Brandmaier P., Seider D., Kolesnik M., Jenniskens S., Sequeiros R.B., Eibisberger M., Voglreiter P., Flanagan R., Mariappan P. (2017). A Prospective Development Study of Software-Guided Radio-Frequency Ablation of Primary and Secondary Liver Tumors: Clinical Intervention Modelling, Planning and Proof for Ablation Cancer Treatment (ClinicIMPPACT). Contemp. Clin. Trials Commun..

